# Plant salt response: Perception, signaling, and tolerance

**DOI:** 10.3389/fpls.2022.1053699

**Published:** 2023-01-06

**Authors:** Fei Xiao, Huapeng Zhou

**Affiliations:** ^1^ Xinjiang Key Laboratory of Biological Resources and Genetic Engineering, College of Life Science and Technology, Xinjiang University, Urumqi, China; ^2^ Key Laboratory of Bio-resource and Eco-environment of Ministry of Education, College of Life Sciences, Sichuan University, Chengdu, China

**Keywords:** salt stress, salt perception, salt response and tolerance, osmoregulation, ionic homeostasis, hormone mediation, light signaling, plant microbiota

## Abstract

Salt stress is one of the significant environmental stressors that severely affects plant growth and development. Plant responses to salt stress involve a series of biological mechanisms, including osmoregulation, redox and ionic homeostasis regulation, as well as hormone or light signaling-mediated growth adjustment, which are regulated by different functional components. Unraveling these adaptive mechanisms and identifying the critical genes involved in salt response and adaption are crucial for developing salt-tolerant cultivars. This review summarizes the current research progress in the regulatory networks for plant salt tolerance, highlighting the mechanisms of salt stress perception, signaling, and tolerance response. Finally, we also discuss the possible contribution of microbiota and nanobiotechnology to plant salt tolerance.

## Introduction

**Figure 1:**
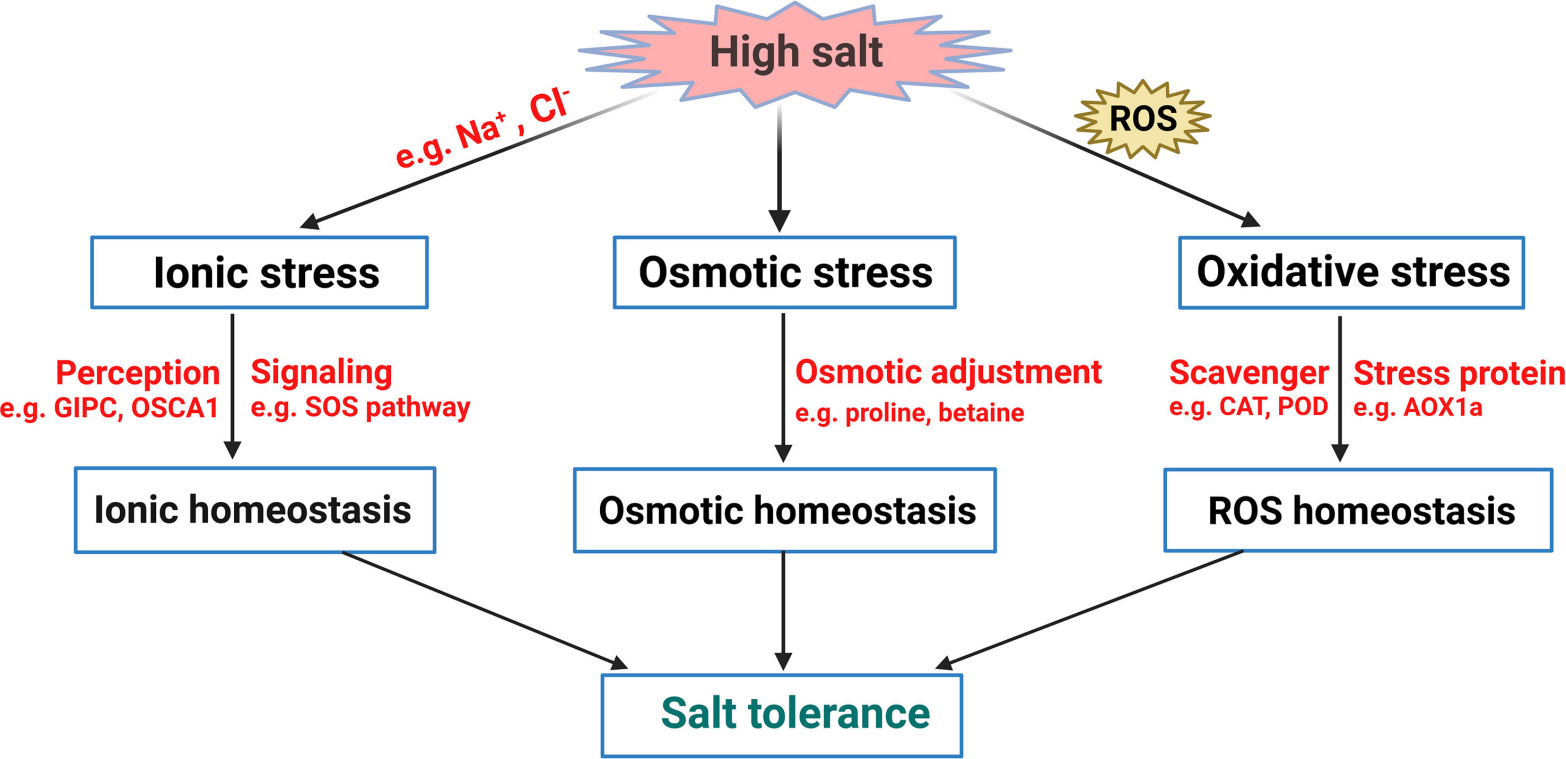
A simplified model of plant salt stress response. Salt stress primarily causes osmotic stress, oxidative stress, and ionic stress. By sensing such stresses, plants activate effective stress signaling networks to accumulate substances for osmotic adjustment, and to maintain ionic and redox homeostasis, leading to salt tolerance in plants.

Soil salinization is one of the most adverse environmental stressors, severely limiting plant growth and development and threatening agricultural production worldwide. In addition to naturally occurring soil salinization, the situation is even exacerbated by excessive chemical fertilizers and soil amendments, improper irrigation practices, and the effect of seawater exposure (Munns and Tester, 2008; Sahab et al., 2021). It is estimated that crop production on at least 20% of global irrigated land is impaired. About 2 million ha (~1%) of world agricultural land is under accelerating salinization each year (Tuteja, 2007). Salt stress causes plant growth inhibition, abnormal development, and metabolic disturbance (van Zelm et al., 2020). The detrimental effects of elevated salinity on plants include (i) osmotic stress as sodium accumulates in the soil and (ii) ionic stress (**Figure 1**) (Yang and Guo, 2018a; Yang and Guo, 2018b; Ludwiczak et al., 2021). Osmotic stress caused by hyperosmotic soil solution disrupts plant cell turgor. In contrast, ionic stress is characterized by disordered sodium (Na^+^)/potassium (K^+^) balance inside the cell, disrupting various metabolic and physiological processes (Zhang et al., 2018). These two processes were reported to be temporally and spatially separated in plants during the salt stress response (van Zelm et al., 2020). However, there exists a significant overlap between osmotic and ionic stress in both early and downstream signaling (Geng et al., 2013; van Zelm et al., 2020). In addition, the elevated content of reactive oxygen species (ROS) in plants is also observed upon salt exposure (**Figure 1**) (Yang and Guo, 2018a). Electrons leaked from the electron transport chain (ETC) can react with O_2_ during aerobic metabolism to produce ROS (Møller, 2001). The toxic level of ROS seriously disrupts normal cellular metabolism through the oxidation of macromolecules like lipids, proteins, and nucleic acids, resulting in severe oxidative damage (Apel and Hirt, 2004; Miller et al., 2010).

In general, there are two plant types regarding their distinct salt tolerance. Some plants, termed halophytes, naturally grow in or even adapt to the saline environment with more than 200 mM NaCl (Duarte et al., 2014). By contrast, most other plant species are salt-sensitive glycophytes, and their growth and development are adversely inhibited by soil salinization (Assaha et al., 2017). Due to their sessile lifestyle, plants have evolved with sophisticated but effective strategies with considerable plasticity in morphology, physiology and metabolism to deal with multiple environmental stimuli (Genoud and Métraux, 1999; Fraire-Velázquez et al., 2011). Therefore, a series of signaling pathways have been established in plants in response to salt stress, including the sensory mechanisms, the networks that mediate osmotic adjustment, redox balance and ionic homeostasis, and other stress response mechanisms (**Figure 1**) (Zhao et al., 2021). In this review, we briefly summarize the new findings of salt stress responses in plants, focusing on recent advances in salt-induced signal perception and transduction. Phytohormone and light signal are essential for plant development and stress response, and some of their regulatory elements in have been also found to play essential roles in coordinating salt stress response in plants (van Gelderen et al., 2018; Kovacs et al., 2019; Waadt et al., 2022). Here we also summarize the mechanisms of phytohormone and light signal-mediated salt tolerance of plants. Finally, we discuss the possible roles of plant microbiota in plant salt tolerance. Understanding the mechanisms of plant salt tolerance provides new opportunities for engineering salt-tolerant plants even grown in saline regions, making it a promising way to keep agricultural productivity and ecological security worldwide.

## Perception of salt stress in plants

**Figure 2:**
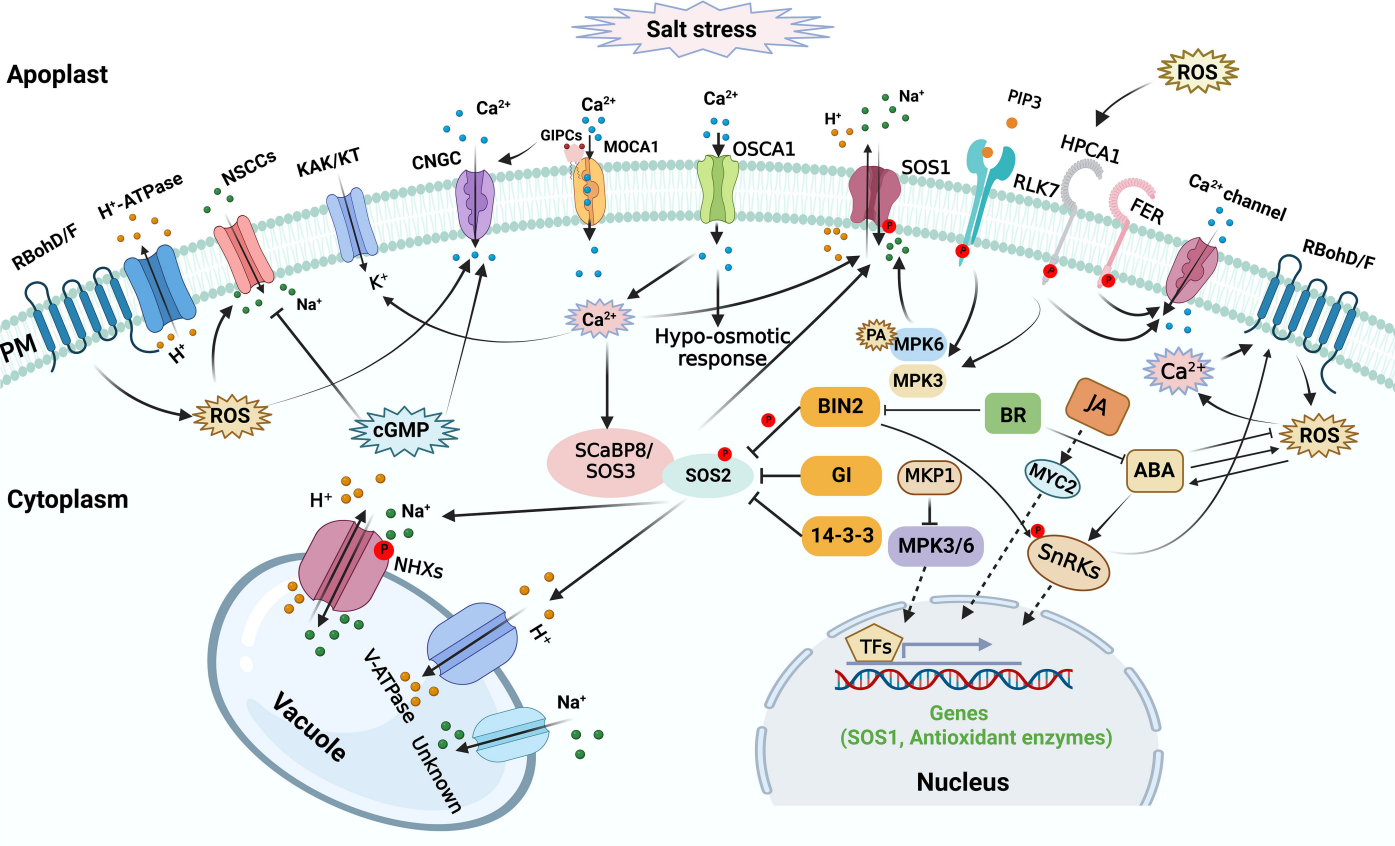
Salt stress sensing and signaling in plant cells. Osmotic alternation and Na^+^ import trigger a rise of cytosolic secondary messengers, which are sensed by specific sensors or receptors, therefore multiple signaling pathways involved in a variety of components are activated to maintain ionic balance and osmotic homeostasis or to regulate osmotic stress response. The arrows and bars indicate positive and negative regulation, whereas solid lines and dashed lines indicate direct regulation and indirect regulation, respectively.

Plants might sense the alteration of salt concentration in the extracellular space and the change of mechanical effects on cellular structures caused by salt stress, triggering a series of signal transduction processes in plants (**Figure 2**). The initial salt stress signals mainly include excess apoplastic Na^+^, cytosolic Ca^2+^ ([Ca^2+^]_cyt_) level increase, ROS accumulation, and 3,5-cyclic guanosine monophosphate (cGMP) production (Shabala et al., 2015; Park et al., 2016a). Under saline condition, excess Na^+^ enters plant roots through nonselective cation channels (NSCCs), which mediate toxic sodium (Na^+^) influx into the cell across the plasma membrane (PM) (Demidchik and Tester, 2002; Demidchik and Maathuis, 2007). Cyclic nucleotide-gated channels (CNGCs) are the main NSCCs, which are implicated in ionic homeostasis during salt response. The initial salt-induced signals might contribute to regulating NSCCs in plants during salt response. NSCCs can be blocked by Ca^2+^ (Leng et al., 2002; Demidchik and Maathuis, 2007; Demidchik et al., 2018). The apoplastic Ca^2+^ concentration in root cells is probably in the region of 0.2-0.4 mM (Legué et al., 1997), which is enough to reduce NSCCs-mediated flux by 30-50% (Essah et al., 2003). The remaining flux can be further diminished by channel blockers like Gd^3+^ and La^3+^ (Demidchik and Maathuis, 2007). Other channels and transporters may also contribute to salt stress perception, but their regulatory role in sodium sensing and import *in planta* is debated (van Zelm et al., 2020; Wang et al., 2022).

Calcium signal functions as an essential secondary messenger, and salt stress leads to rapid and transient [Ca^2+^]_cyt_ elevations to trigger salt tolerance responses (Knight et al., 1991; Knight et al., 1997). During initial salt response, calcium signal plays a vital role in osmosensing within seconds of exposure to osmotic stress caused by the saline conditions. REDUCED HYPEROSMOLALITY- INDUCED [Ca^2+^]_cyt_ INCREASE1 (OSCA1) was initially identified as a hyperosmolality-activated Ca^2+^-permeable cation channel responsible for [Ca^2+^]_cyt_ increase, might be a potential osmosensor in plants (Yuan et al., 2014). A Ca^2+^-responsive phospholipid-binding BONZAI (BON) protein was recently reported to mediate hyperosmotic stress tolerance by positively regulating osmotic stress-induced [Ca^2+^]_cyt_ increase, ABA accumulation, and gene expression, indicating a possible role of the membrane-associated Ca^2+^-responsive BON proteins in osmotic sensing and signaling (Chen et al., 2020). Considering that salt stress triggers osmotic stress, it is possible that the aforementioned Ca^2+^ transporters and Ca^2+^-responsive BON protein might participate in initial salt sensing and signaling by mediating Ca^2+^ signal in plants. Recently, the *Arabidopsis thaliana* (Arabidopsis) PM glycosyl inositol phosphorylceramide (GIPC) sphingolipids was found to function as a sensor to sense Na^+^ level and regulate salt stress response by gating ionic stress-induced Ca^2+^ signaling (Jiang et al., 2019). GIPC sphingolipid biosynthesis is catalyzed by the protein of monocation-induced Ca^2+^ increases1 (MOCA1), and extracellular Na^+^ ions can bind GIPC sphingolipids to gate PM Ca^2+^ influx channels. Consistently, the *moca1* mutant is hypersensitive to salt stress and lacks cation-evoked Ca^2+^ spikes and waves (Jiang et al., 2019).

High salinity stress rapidly triggers H_2_O_2_ bursts in plant cells, which might function as essential salt stress signal (Wang et al., 2020). HYDROGEN-PEROXIDE-INDUCED CA^2+^ INCREASES1 (HPCA1) functions as a H_2_O_2_ sensor that perceives the stress-induced extracellular H_2_O_2_ burst and generates increased [Ca^2+^]_cyt_ under stress stimuli (Wu et al., 2020). Mathematical modeling showed that under salt stress, crosstalk between ROS and Ca^2+^ signaling is necessary to spread the Ca^2+^ signal between cells (Evans et al., 2016). Accordingly, the *atrbohd atrbohf* double mutant, which is hypersensitive to salt stress, exhibited a reduction in cytosolic free Ca^2+^ and PM Ca^2+^ influx (Ma et al., 2012). Importantly, Jiang et al., showed that lack of AtrbohF causes hypersensitivity of shoots to soil salinity with high Na^+^ accumulation in root vasculature cells and xylem sap (Jiang et al., 2012). AtrbohF-mediated ROS production in root vasculature contributes to Na^+^ concentration limitation, thus protecting shoot cells from transpiration-dependent excess Na^+^ delivery (Jiang et al., 2012). Moreover, the Ca^2+^ signaling complex CBL1/9-CIPK26 interacts with and phosphorylates AtRbohF (Drerup et al., 2013). Thus, in the early stages of salt stress, ROS and Ca^2+^ signals work together to affect ionic homeostasis in plants.

The rise of cellular cGMP can be detected within seconds after applying salinity and osmotic stress (Donaldson et al., 2004). Furthermore, cGMP inhibits Na^+^ influx in several plant species (Maathuis and Sanders, 2001; Essah et al., 2003; Rubio et al., 2003), while it can regulate transcription of various genes related to salinity stress and promote K^+^ uptake (Maathuis, 2006; 2014; Isner and Maathuis, 2016). Importantly, cGMP has a negative effect on the inward Na^+^ flux acrried by NSCCs (Maathuis and Sanders, 2001).

Peptide ligands have also emerged as essential mediators of cell-to-cell communication during plant growth and stress responses, possibly through the action of their PM-localized receptors, RECEPTOR-LIKE KINASEs (RLKs) (Gancheva et al., 2019; Xie et al., 2022). For instance, the defense-related peptide PLANT ELICITOR PEPTIDE AtPep3 and its receptor PEP1 RECEPTOR 1 (PEPR1) are implicated in plant salt response, and PEPR1 loss of function nearly abolishes AtPep3-induced salt resistance in Arabidopsis (Nakaminami et al., 2018). Recently, Zhou et al., reported that the RECEPTOR-LIKE KINASE 7 (RLK7), another PM receptor kinase in Arabidopsis, can be recognized by a secreted peptide, PAMP-INDUCED SECRETED PEPTIDE 3 (PIP3), and form an active ligand-receptor signaling cascade that modulates plant salt tolerance in Arabidopsis by activating MPK3/MPK6 cascade (Zhou et al., 2022). Furthermore, LEUCINE-RICH REPEAT EXTENSINs, RAPID ALKALINIZATION FACTOR peptides and FERONIA (FER), a member from the *Catharanthus roseus* RLK1-like (*Cr*RLK1L) family in Arabidopsis, may form a functional module that connects salt stress-induced cell-wall alterations to salt stress responses (Zhao et al., 2018). In addition, FER-dependent signaling may elicit a cell-specific Ca^2+^ signal to maintain cell wall integrity during salt stress and root growth recovery after salt exposure (Feng et al., 2018). Although some peptides are implicated in plant salt response, upregulation of their coding genes and the maturation of these peptides during salt stress response might depend on the initial signals such as Ca^2+^.

## Salt responses in plants

### Osmotic adjustment

The immediate problem plants face is dehydration due to osmotic stress caused by high salinity, and plants can initiate osmotic adjustment to maintain cell volume and turgor under salt stress (**Figures 1**, **2**) (van Zelm et al., 2020). The osmotic adjustment is a process by which plants enhance their water availability by synthesizing compatible solutes, known as osmolytes, in their cells. The osmolytes mainly include organic substances and inorganic ions, and proline, glycine betaine, and soluble carbohydrates, have been proven effectively regulate osmotic pressure by lowering the osmotic potential in the cytosolic compartment, thus preventing cellular dehydration during salt stress (Yang and Guo, 2018a; Jogawat, 2019). Due to its strong hydration ability, proline accumulation is an effective strategy to prevent protein dehydration and denaturation under osmotic stress in plants (Liang et al., 2013). Data showed that treatment with proline to two contrasting cultivars of *Brassica juncea* could alleviate the adverse effects of salinity on photosynthesis and seed yield (Wani et al., 2019). Furthermore, proline can function as an O_2_ quencher, thus removing excessive ROS produced under stress (Rejeb et al., 2014; Rehman et al., 2021). Glycine betaine, a water-soluble amphoteric quaternary ammonium compound, is also an essential osmotic regulator in higher plants, playing a vital role in stress alleviation (Ashraf and Foolad, 2007). Exogenous application of glycine betaine can mitigate salt-induced damage in maize seedlings (Hossain et al., 2021; Bai et al., 2022). Non-structural carbohydrates, such as glucose, sucrose, fructan, and starch, are also found to accumulate in plants under salt stress and function in osmotic adjustment, therefore enhancing the salt tolerance of plants (Munns and Tester, 2008; Li and Sheen, 2016; Wang et al., 2021). Significantly, carbohydrates also act as sugar signaling in plant response to an environmental stimulus (Bhattacharya and Kundu, 2020), making it another layer of regulation in plant salt tolerance. Apart from organic solutes, inorganic ions have been demonstrated as significant contributors to osmotic adjustment during saline conditions. Sodium and chloride are toxic to plants, but salt-tolerant plants might take them as essential osmolytes to maintain the external osmolarity to avoid osmotic imbalance and sustain growth (Munns and Tester, 2008; Tanveer and Shah, 2017; Hussain et al., 2021). Plants enhance their environmental adaptability by accumulating inorganic ions like K^+^ under salt stress. It has been revealed that K^+^ is an influential contributor to osmotic regulation (Wang et al., 2013; Kumari et al., 2021), making it a crucial element for plant growth and stress response.

### ROS homeostasis and redox regulation

ROS in plant cells mainly includes oxygen radicals, like superoxide (^*^O_2_^-^), hydroxyl radical (^*^OH), and some non-radicals, such as hydrogen peroxide (H_2_O_2_), singlet oxygen (^1^O_2_), and ozone (O_3_) (Gill and Tuteja, 2010). The generation sites of ROS in salt-stressed plants mainly include chloroplasts, mitochondria, apoplast, and peroxisomes (Sharma et al., 2012). ROS might damage cells at excess levels while act as crucial signaling molecules essential for stress signaling at lower concentrations (**Figure 2**) (D'Autréaux and Toledano, 2007; Sewelam et al., 2016). The interaction between ROS and ethylene (ET) has been identified as the primary signal that mediates salinity stress in rice (Steffens, 2014). Excessive accumulation of ROS induced by salt stress is toxic to plants and causes oxidative damage to cellular constituents, leading to cell death (Ahanger et al., 2017; Ye et al., 2021). A high level of ROS (especially H_2_O_2_) leads to DNA damage and distorts genomic stability (**Figure 1**) (Lin et al., 2020). To detoxify ROS generated by salt stress, plants have evolved a set of antioxidant strategies, mainly including enzymatic (Racchi, 2013) and non-enzymatic (Khazaei and Aghaz, 2017) systems to protect cells from oxidative damage (**Figure 1**). Enzymatic systems include a set of superoxide dismutase (SOD), peroxidase (POD), catalase (CAT), and ascorbate peroxidase (APX). Numerous studies have shown that salt-tolerant species improve their salt tolerance by enhancing the antioxidant defense system under salt stress (Souana et al., 2020; Challabathula et al., 2022). Non-enzymatic antioxidants, such as glutathione (GSH), ascorbate (AsA), monodehydroascorbate reductase (MDHAR), and dehydroascorbate reductase (DHAR), can effectively scavenge some highly toxic ROS in plants (Maurya, 2020). Data showed that GSH and AsA levels increase with an elevated level of ^*^O_2_^-^ and H_2_O_2_ in *Camellia sinensis* (L.) exposed to 300 mM NaCl stress (Li et al., 2019). Enhanced activities of MDHAR and DHAR enzymes were reported to be induced by salt stress, thus decreasing cell membrane damage in sugar beet M14 (Li et al., 2020a). Consequently, plant antioxidant defense is proficient in maintaining ROS balance for salt tolerance. Importantly, as the interaction between O_2_ and reduced ETC components leads to ROS production, one effective strategy to reduce toxic ROS level is to prevent ETC over-reduction (Maxwell et al., 1999). The mitochondrial alternative oxidases (AOXs) participate in electron overflow when the cytochrome ETC chain is saturated with electrons due to impaired electron transport under challenging conditions, preventing further reduction of ubiquinone and stabilizing the whole ETC (Millenaar et al., 1998; Vanlerberghe, 2013). Our recent work demonstrates that SIZ1-mediated SUMOylation of R2R3-MYB transcription factor MYB30 modulates plant salt tolerance through the action of AOX1a. MYB30 binds the promoter of *AOX1a* and upregulates its expression in response to salt stress to maintain the cellular redox homeostasis through enhanced alternative respiration pathway (Gong et al., 2020).

### Ionic balance regulation

Under salinity stress, excess toxic sodium ions (Na^+^) enter and accumulate in plant cells, disrupting ion homeostasis, especially Na^+^/K^+^ balance (**Figures 1**, **2**) (Munns and Tester, 2008; Zhao et al., 2021). Keeping ionic homeostasis is a prerequisite for plant growth during salt stress, since disordered ionic homeostasis leads to the disruption of cellular metabolism (Zhu, 2003; Amin et al., 2021). Plants have developed sophisticated and effective mechanisms to keep optimal levels of Na^+^ by removing or vacuolar compartmentalizing Na^+^ from the cytoplasm, and a variety of carrier and channel proteins, symporters, and antiporters participate in this process (**Figures 1**, **2**) (Tester and Davenport, 2003; Munns and Tester, 2008; Wu, 2018). On the other hand, K^+^ retention in the cytosol is esstisal for Na^+^/K^+^ balance during salt response (Yang et al., 2014; Yang and Guo, 2018a). The Na^+^/H^+^ antiporters that transport Na^+^ in exchange for H^+^ achieve the regulation of Na^+^ levels in the cytoplasm in plant cells. The PM-localized Na^+^/H^+^ antiporters can transport Na^+^ to the apoplast, and the vacuole-localized Na^+^/H^+^ antiporters are responsible for vacuolar sequestration of Na^+^ (Qiu et al., 2004; Keisham et al., 2018; Akhter et al., 2022). The increased accumulation of Na^+^ in vacuoles might also act as an osmoticum, enhancing salt tolerance ability (Solis et al., 2021). NHXs are putative Na^+^/H^+^ exchangers that transport Na^+^ from the cytoplasm to the vacuole, holding plant resistance to salt stress (Yokoi et al., 2002; Su et al., 2020). Several reports showed that overexpression of *NHX* confers salinity tolerance in many plant species. For example, constitutive overexpression of *AtNHX1* significantly increases salt tolerance in rice (Fukuda et al., 2004), wheat (Xue et al., 2004), tomato (Zhang and Blumwald, 2001), and cotton (He et al., 2005). Taken together, Na^+^ exclusion, vacuolar Na^+^ sequestration, and K^+^ retention in the cytosol are essential for plant salt tolerance (**Figure 2**).

The Salt Overly Sensitive (SOS) regulatory pathway plays a pivotal role in regulating ionic homeostasis through modulating the activity of Na^+^/H^+^ antiporters under salt stress (**Figure 2**) (Yang et al., 2009; Ji et al., 2013). The SOS pathway effectively maintains the Na^+^ homeostasis by transporting excess Na^+^ from the cytosol to the apoplast, thus preventing the accumulation of Na^+^ to toxic levels (Halfter et al., 2000; Yang et al., 2009; Quintero et al., 2011). The SOS signaling pathway includes Salt Overly Sensitive-1 (SOS1), a PM Na^+^/H^+^ antiporter, the serine/threonine protein kinase SOS2, and two calcium sensors, SOS3 and SCaBP8/CBL10 (SOS3-like calcium-binding protein 8) (Yang and Guo, 2018a). When grown in normal condition, SOS pathway is “off” *via* the action of 14-3-3 proteins and GIGANTEA (GI) which interact with SOS2 and repress its kinase activity (Kim et al., 2013; Zhou et al., 2014). High salinity initiates a calcium signal that activates the SOS pathway (**Figure 2**). Under salt stress, 14-3-3 proteins are released from SOS2 and degraded through proteasomal pathways (Tan et al., 2016); SOS3/SCaBP8 protein perceives the increased [Ca^2+^]_cyt_, recruits SOS2 to the PM, and activates its activity (Halfter et al., 2000; Quan et al., 2007; Lin et al., 2009). Subsequently, the activated SOS2 phosphorylates SOS1, thus enhancing the transport activity of SOS1 and transporting Na^+^ from cytosol to apoplast (Quintero et al., 2011). In addition, SOS3/SCaBP8-SOS2 module might also positively regulate the activities of other transporters involved in ionic homeostasis like vacuolar Na^+^/H^+^ exchanger (NHX) (Zhao et al., 2021). Remarkably, SOS1-mediated Na^+^ exclusion in plants during salt response is regulated by MITOGEN-ACTIVATED PROTEIN KINASE (MAPK) signaling pathways (**Figure 2**). For instance, MITOGEN-ACTIVATED PROTEIN KINASE3 (MPK3) and MPK6 physically interact with and phosphorylates SOS1 and salt stress-induced PA (Yu et al., 2010), and thus mediate salt response and suppresse Na^+^ accumulation *via* in shoots (Ji et al., 2013). Phosphatase MAP KINASE PHOSPHATASE1 (MKP1) can be phosphorylated by MPK6 (Park et al., 2011), and MKP1 exhibits a negative effect on MPK3/6 activity (Bartels et al., 2010; Besteiro et al., 2011). A recent study revealed that the *mkp1* mutation improves salt tolerance by restraining Na^+^ accumulation in shoots (Uchida et al., 2022). The salt tolerance in *mkp1* might be attributed to the activation of SOS1 *via* the elevation of MPK6.

Besides *Arabidopsis thaliana*, *SOS* genes have been identified in many other plants, such as *Triticum aestivum L.* (Jiang et al., 2021) and *Oryza sativa* (Kumar et al., 2012). Overexpression of *SOS* genes could improve salt tolerance by regulating ionic homeostasis (Baghour et al., 2019; Gupta et al., 2021). Importantly, it is worth noting that the entry of Na^+^ across the tonoplast membrane or PM is driven by the proton motive force established by proton pumps in the tonoplast or PM. Activation of H^+^-ATPase (Conde et al., 2011) and H^+^ pyrophosphatases (Maeshima, 2000) generates such proton motive force across the PM, thus activating most of the ion and metabolite transport. Vacuolar H^+^-ATPase (V-ATPase) is the most prevailing H^+^ pump in the plant cells (Dietz et al., 2001). Studies have revealed that enhancing the expression level of V-ATPase could improve salt tolerance (Zhang et al., 2012). Interestingly, both V-ATPases and PPase are also thought to be regulated by the SOS components (Batelli et al., 2007; Silva and Gerós, 2009).

Under salinity stress, excessive Na^+^ leads to K^+^ loss in plant cells (Park et al., 2016a; Zhao et al., 2021). The transporters HAK/KT/KUP play an essential role in maintaining Na^+^/K^+^ homeostasis during salt stress, which is involved in enhancing K^+^ absorption and reducing Na^+^ accumulation inside the cells (Almeida et al., 2017). In rice, OsHAK1 dominates the Na^+^-sensitive high affinity K^+^ uptake system (Chen et al., 2017). Constitutive expression of OsHAK5 in BY2 cells enhances K^+^ accumulation under saline condition and confers salt tolerance in these cells (Horie et al., 2011; Yang et al., 2014). OsHAK5 might mainly function in shoot tissues and its overexpression leads 43-115% increase in K^+^/Na^+^ ratio compared to WT plants in shoot but not root (Yang et al., 2014). OsHAK21 is also reported to mediate K^+^ absorption across the PM and play an essential role in maintaining the Na^+^/K^+^ homeostasis in rice under salt stress (Shen et al., 2015). The mutant of *oshak21* accumulates less K^+^ and considerably more Na^+^ in both shoots and roots compared with the wild type. Research also suggests that Arabidopsis NADPH oxidases ARABIDOPSIS THALIANA RESPIRATORY BURST OXIDASE HOMOLOG D (AtrbohD) and AtrbohF function in ROS-dependent regulation of Na^+^/K^+^ homeostasis under salinity stress possibly regulating inward K^+^ currents under both normal and salt stress conditions (Ma et al., 2012).

## Phytohormone signaling and plant salt tolerance

**Figure 3:**
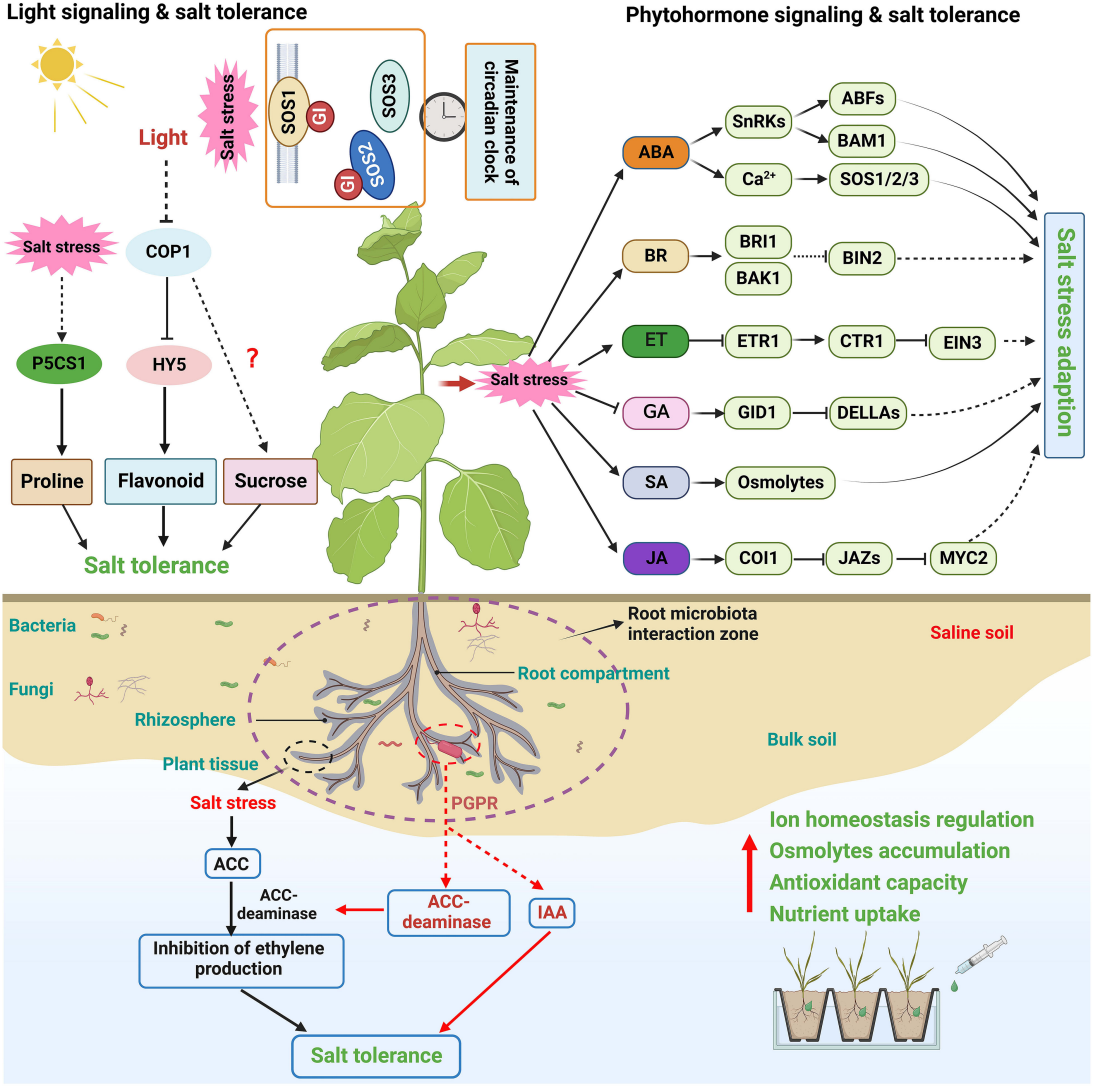
Schematic diagram of phytohormone, light signal, and microbiota-mediated plant salt tolerance. Phytohormone and light signal are essential for plant development and stress response, and some of their regulatory elements in have been also found to play essential roles in coordinating salt stress response in plants. Plant hormone signaling plays integrated and sophisticated roles at different vegetative stages, in different tissues, or under various environmental stimuli. Light signaling networks in plants begin with the perception of light signals, and are vital in shaping plant salt stress response. Plant microbiota plays a vital role in plant adaptation and resistance to saline soil. PGPR is essential to improve plant tolerance to high salinity, possibly by regulating ionic homeostasis, accumulating osmolytes, activating antioxidant capacity, and enhancing essential nutrient uptake.

Phytohormones are small chemicals that play an essential role in plant growth and development. Evidence indicates that phytohormones mediate various stress resistance, such as salt, osmotic, drought, cold, and pathogen stress (Carvalho et al., 2013; Verma et al., 2016; Yu et al., 2020; Waadt et al., 2022). Numerous studies have shown that plant hormone signaling plays integrated and sophisticated roles at different vegetative stages, in different tissues, or under various environmental stimuli (Ku et al., 2018; Waadt et al., 2022). How plant hormones, including abscisic acid (ABA), brassinosteroid (BR), ethylene (ET), gibberellin (GA), salicylic acid (SA), and jasmonic acid (JA), mediate salinity signals to regulate plant salt stress tolerance is briefly summarized here (**Figure 3**).

### ABA signaling

ABA functions as an essential central integrator to activate adaptive signaling cascades during the salt stress response in plants. Under abiotic stresses, including salinity and water deficit, endogenous ABA levels increase rapidly, and enhanced ABA signaling activates sucrose nonfermenting 1-related protein kinases (SnRK2s) (Zhu, 2016). SnRK2s are the central components in ABA signaling networks and play critical roles in ion transport, osmoregulation, ROS production, gene transcription, and the closing of stomata (Yang G et al., 2019). Stomata are the primary place for plant transpiration, and ABA-regulated stomatal opening and closing are critical for plants to respond to salt stress. OST1/SnRK2.6 interacts with and phosphorylates specific ion channels, such as the potassium channel KAT1 and the slow anion channel SLAC1, to mediate K^+^ efflux and anion currents in guard cells, thus enhancing stomatal closure during salt and osmotic stress (Brandt et al., 2015). SnRK2.2/2.3/2.6 phosphorylate and positively control various ABA-responsive element (ABRE)-binding protein/ABRE-binding factor (AREB/ABF) transcription factors, further regulating osmotic stress response in plants (Cai et al., 2017). ABA-activated SnRK2s also regulate osmotic stress tolerance by controlling the BAM1- and AMY3-dependent degradation of starch into sugar and sugar-derived osmolytes (Thalmann et al., 2016). It is deserved to determine whether similar mechanisms are involved in the osmotic regulation during salt stress in plants.

Salt stress leads to the increase of [Ca^2+^]_cyt_ (Yang Y et al., 2019). Ca^2+^ signaling plays a real-time and influential role in response to salinity stress. ABA effectively helps plants survive salt stress by integrating with the versatile second messenger Ca^2+^ *via* provoking PM-bound channels or releasing Ca^2+^ from intracellular Ca^2+^ pools (Edel and Kudla, 2016). The damage to the cell wall caused by Na^+^ can be perceived by the kinase FER, which mediates salt stress signaling by increasing [Ca^2+^]_cyt_. In contrast, ABA flexibly controls FER activity through the dephosphorylation of ABA INSENSITIVE 2 (ABI2) (Chen et al., 2016). In addition, ABA-activated SnRKs can phosphorylate the membrane-bound NADPH oxidase AtrbohF, modulating ROS homeostasis in plant response to high salinity (Szymańska et al., 2019). A recent study has demonstrated that salt-induced ABA and Ca^2+^ signaling can fine-tune AtrbohF activity by activating SnRK2.6 and CIPK11/26 signaling modules (Han et al., 2019). Together, ABA, Ca^2+^ and ROS exhibit complicated signaling crosstalk to control plant resistance to salt stress.

### BR signaling

BRs are a class of steroid phytohormones in plants and play pivotal roles in plant growth, development, and response to adverse stresses (Ahanger et al., 2018; Nolan et al., 2020). BRs have been widely reported to improve salt stress tolerance in a range of plants, including Arabidopsis, rice (*Oryza sativa*), tomato (*Lycopersicon esculentum*), and mustard (*Brassica napus*) (Özdemir et al., 2004; Wani et al., 2019; Jia et al., 2021). BR can significantly inhibit ROS generation by enhancing antioxidant capacity under saline conditions (Fariduddin et al., 2013; Li S. et al., 2020). Exogenous treatment with 24-epibrassinolide (EBL), an active by-product from brassinolide biosynthesis, effectively improves salt tolerance in soybean through regulating enzymatic antioxidants and osmolyte accumulation (Soliman et al., 2020). The exogenous application of BR has been reported to improve photosynthetic efficiency in different plant species. In a recent study, EBL application could alleviate the detrimental effects of salt stress on chloroplasts and photosynthesis in *Robinia pseudoacacia* L. seedlings (Yue et al., 2019). Moreover, exogenous BR application could also relieve salt toxicity by regulating the activity of Na^+^/H^+^ antiporters and NHX (Su et al., 2020). These results highlight the potential role of BR in plant salt resistance.

BR-induced enhanced tolerance to salinity is closely associated with BR signaling. When extracellular BR hormones directly bind to one of its membrane-localized receptors, BRASSINOSTEROID-INSENSITIVE 1 (BRI1), BRI1-LIKE 1 (BRL1) or BRL3, and the coreceptor BAK1(SERK3) in Arabidopsis, an efficient phosphorylation cascade, to relay BR signals to BRI1-EMS-SUPPRESSOR1 (BES1) and BRASSINAZOLE-RESISTANT1 (BZR1) family TFs, therefore controlling BR-regulated gene expression (He et al., 2000; Nolan et al., 2020). Salt stress leads to root growth inhibition in plants due to a reduced level of BZR1 in the nucleus and the repression of BR signaling (Srivastava et al., 2020). However, exogenous BR application can even partially enhance salt-induced growth inhibition (Liu et al., 2014; Guedes et al., 2021). Studies indicate that overexpression of vascular BR receptor BRL3 promotes the accumulation of osmoprotectant metabolites, including proline and sugars, which play essential roles in osmoregulation under salt stress (Fàbregas et al., 2018). Overexpression of SERK2, an interacting partner of BR receptor in rice, significantly enhances grain size and salt stress resistance (Dong et al., 2020). The accumulation of SERK2 induced by salt stress confers early BR signaling on the PM to enhance the salt stress response. SlBZR1, a BZR/BES TF in tomatoes, positively regulates BR signaling and salt stress tolerance in tomatoes and Arabidopsis (Jia et al., 2021). BIN2 is a critical negative component of BR signaling, which also acts as an essential molecular switch to balance plant growth recovery and salt stress response in Arabidopsis; however, BR signaling might not be implicated in BIN2-SOS2 module during salt response and growth recovery regulation (Li et al., 2020b). Importantly, multilayer crosstalks between BR and ABA have been observed. BIN2 activates ABA signaling through the phosphorylation of SnRK2.2 and SnRK2.3 (Cai et al., 2014). In contrast, phosphatases ABI1 and ABI2, two major negative players in ABA signaling, can mediate the dephosphorylation of BIN2, abolishing the activity of BIN2 and enhancing the transduction of the BR signaling pathway (Wang et al., 2018). It is possible that BR and ABA together control plant growth and salt stress response

### Other phytohormone signaling pathways

ET and GA have been also reported to be involved in plant salt stress response. Plants rapidly generate gaseous ET under salt stress (Zhang et al., 2016). Increased endogenous ET or treatment with ACC, an ET precursor, both can enhance plant salt tolerance (Tao et al., 2015; Gharbi et al., 2017). ET promotes plant salt tolerance by maintaining the homeostasis of Na^+^/K^+^ and reducing ROS by inducing antioxidant defense (Yang et al., 2013; Wang et al., 2020). ET signaling is essential in plant salt tolerance. Loss of function of ET receptor ETHYLENE RESPONSE 1(ETR1) and ETHYLENE INSENSITIVE 4 (EIN4) or CONSTITUTIVE TRIPLE RESPONSE 1 (CTR1), a negative regulator of ET signaling, confers enhancement of salt tolerance (Wilson et al., 2014; Dubois et al., 2018), while loss-of-function mutants of ETHYLENE INSENSITIVE 3 (EIN3) and EIN3-LIKE 1 (EIL1), two ET-activated TFs, exhibited higher salt sensitivity in contrast to wild-type plants (Peng et al., 2014). As a well-known regulator of seed germination, GA positively regulates plant growth (Sun and Gubler, 2004). By contrast, reduced bioactive GA levels or signaling after germination is required for plant tolerance to salt stress (Magome et al., 2004; Magome et al., 2008). Consistently, the GA-deficient mutant *ga1-3* exhibits remarkable tolerance to salt stress (Magome et al., 2004); the growth of seedlings lacking GA signaling repressors GAI, RGA, RGL1, and RGL2 is less inhibited by salt stress compared with the corresponding wild-type plants (Achard et al., 2006; Achard et al., 2008). Moreover, overexpression of the GA catabolic gene *CYP71D8L* improves rice tolerance to salinity stress by affecting GA homeostasis (Zhou et al., 2020). The phytohormone SA is also repeatedly reported to take part in salt tolerance. For example, the exogenous application of SA together with nitric oxide (NO) significantly alleviates the NaCl-mediated oxidative damage in *Vigna angularis* by enhancing the synthesis of osmotic substances and improving photosynthesis (Ahanger et al., 2019). A recent study showed that priming the seed germination of *Leymus chinensis* in SA solution relieves salt-induced osmotic damage by accumulating K^+^ (Hongna et al., 2021). It might be due to the improved ATP content and H^+^-ATPase activity in the membrane of root cells (Ghassemi-Golezani and Farhangi-Abriz, 2018). In addition, SA might also crosstalk with other hormones, such as ABA, ET, and GA, which are closely correlated with the activation of osmotic adjustment and maintenance of ionic homeostasis (Khan et al., 2014; Jayakannan et al., 2015).

As a stress-related hormone, JA has been also found to be involved in salt-induced growth inhibition (Valenzuela et al., 2016). Salt stress induces the expression of JA biosynthesis-related genes in leaves and roots, leading to increased JA production (Du et al., 2013; Delgado et al., 2021). Exogenous application of JA significantly alleviates salt-induced damage by increasing the antioxidative enzyme activities and maintaining Na^+^/K^+^ balance (Qiu et al., 2014; Gao et al., 2021). JA signaling plays an essential role in plant salt tolerance. The crucial component activating JA signaling, MYC2, is implicated in salt-mediated JA-dependent inhibition of cell elongation in the elongation zone of Arabidopsis primary roots (Valenzuela et al., 2016; Verma et al., 2020). Additionally, MYC2 contributes to salt tolerance by regulating the proline biosynthesis gene in Arabidopsis (Verma et al., 2020). Jasmonate ZIM-domain (JAZ) proteins are the core components of the JA signaling pathway, and their roles in plant salt stress response have been characterized in many species. A recent study has shown that GaJAZ1 interacts with GaMYC2 to inhibit the expression of downstream genes, increasing salt tolerance in *Gossypium hirsutum* (Zhao et al., 2020). On the other hand, overexpression of CYP94C2b, a cytochrome P450 family protein involving JA catabolism, enhanced viability under salt conditions and delayed the salt stress-induced leaf senescence in rice (Kurotani et al., 2015).

## Light signaling and plant salt tolerance

The light signaling networks in plants begin with the perception of light signals, ultimately leading to changes in plant development and stress response (van Gelderen et al., 2018). Emerging evidence shows that light signaling is vital in shaping plant salt stress response (**Figure 3**) (Carvalho et al., 2011; Kovacs et al., 2019). For example, light signaling can affect salt stress-induced transcriptional memory response of P5CS1-mediated proline accumulation in Arabidopsis (Feng et al., 2016). In addition, PHYTOCHROME-INTERACTING FACTOR 4 (PIF4), a negative regulator of the phytochrome signaling pathway, negatively regulates plant salt tolerance by downregulating the expression of stress tolerance genes (Leivar and Quail, 2011; Sakuraba et al., 2017). CONSTITUTIVE PHOTOMORPHOGENIC1 (COP1), a master of the light signaling pathway, also regulates salt stress tolerance. Salt treatment can promote the translocation of COP1 to the cytosol; the *cop1* mutants exhibited a significantly impaired resistance to salt stress than the wild-type plants at the germination and seedling stages (Yu et al., 2016). A recent study further confirmed that COP1 controls plant salt stress tolerance by modulating sucrose content (Kim et al., 2022). Moreover, constitutive nuclear-localized ELONGATED HYPOCOTYL 5 (HY5), a bZIP family TF acting as a critical regulator in light signaling and seedling development (Cluis et al., 2004; Gangappa and Botto, 2016), can promote proline biosynthesis by connecting light and salt stress signals (Kovacs et al., 2019). Recently, a study on tobacco suggests that NtHY5 enhances salt stress tolerance by positively regulating light-mediated flavonoid biosynthesis (Singh D et al., 2022).

Circadian clock regulates many physiological and developmental processes in plants, and its phase and period are adjusted by light, temperature, and nutrient input (Greenham and McClung, 2015; Greenwood and Locke, 2020). It has been found that salt tolerance is also regulated by the circadian clock *via* modulating the expression of salt-responsive genes like *RD29A* and *SOS1* (Park et al., 2016b). The protein abundance of PM Na^+^/H^+^ antiporter SOS1 appears to occur in a diurnal cycle (Park et al., 2016b). Interestingly, a recent work revealed that SOS1 specifically functions as a salt-specific circadian clock regulator *via* GI in Arabidopsis. SOS1 directly interacts with GI in a salt-dependent manner and stabilizes this protein to sustain a proper clock period under saline conditions for the homeostasis of the salt response under high or daily fluctuating salt levels (**Figure 3**) (Cha et al., 2022). The regulatory role of light signals in plant salt tolerance needs to be clarified next.

## Microbiota and plant salt tolerance

Plants host a diverse community of microorganisms on and inside organs such as roots and leaves, collectively termed the plant microbiota (Vandenkoornhuyse et al., 2015; Dastogeer et al., 2020). Accumulating evidence indicates that plant microbiota plays a vital role in plant adaptation and resistance to saline soil (Ha-Tran et al., 2021; Chialva et al., 2022). Notably, multiple groups of root-associated microbes, including plant-growth-promoting rhizobacteria (PGPR) and endophytic bacteria, are essential to improve plant tolerance to high salinity (**Figure 3**) (Qin et al., 2016; Vives-Peris et al., 2018). PGPR alleviates the toxicity of salt stress on plants mainly by regulating ionic homeostasis, accumulating osmolytes, activating antioxidant capacity, and enhancing essential nutrient uptake (Santos et al., 2018; Ha-Tran et al., 2021; Shabaan et al., 2022). For instance, a recent study suggested that the bacterial strain *E. cloacae* PM23 mediated salt tolerance in maize by modulating plant physiology, antioxidant defense, and compatible solute accumulation (Ali et al., 2022).

Furthermore, PGPR can produce ACC deaminase, which reduces the excessive ET production in plants caused by salt stress. Plants with reduced ET level would finally cope with salt-induced growth inhibition by associating with ACC deaminase-producing microbes (Glick et al., 2007; Barnawal et al., 2014; Misra and Chauhan, 2020). Studies also indicate that PGPR can improve plant salt tolerance by producing a wide range of phytohormones as signal molecules in the rhizospheric region (Khan et al., 2020; Jalmi and Sinha, 2022). For instance, it has been revealed that an Algerian Sahara PGPR named strain Pp20 confers maize root tolerance to salt stress *via* producing and secreting plant growth-promoting hormone indole-3-acetic acid (IAA) and ACC deaminase (Zerrouk et al., 2019). In addition, the endophytic bacteria penetrating into the plant root cells possess similar functions in improving salt tolerance compared with PGPR (Sgroy et al., 2009; Yaish et al., 2015). For example, ACC deaminase-containing endophytic bacteria can ameliorate salt stress in *Pisum sativum* through reduced oxidative damage and induction of antioxidative defense systems (Sofy et al., 2021). Remarkably, some root-associated fungal endophytes are also shown to improve plant salt tolerance in terms of growth, ion homeostasis, and osmoregulation (Rodriguez et al., 2009; Bouzouina et al., 2021; Moghaddam et al., 2021). Taken together, both rhizospheric and endophytic bacteria can be employed as effective and eco-friendly adjuncts to promote plant tolerance to salinity (**Figure 3**).

## Nanobiotechnology and plant salt tolerance

In recent years, the plant nanobiotechnology approach has shown great potential to modulate plant stress response (Hofmann et al., 2020; Li et al., 2022). Nanotechnology is the application of small-sized materials with a basic structure of 1–100 nm (Farokhzad and Langer, 2009). A variety of nanomaterials (NMs) have been reported to enhance plant salt tolerance for growth under saline condition (Almutairi, 2016; Zulfiqar and Ashraf, 2021). For instance, some metal-based nanoparticles, cerium oxide nanoparticles, silica nanoparticles, titanium dioxide nanoparticles, and zinc oxide nanoparticles, can improve salt resistance in multiple plant species (Newkirk et al., 2018; Gaafar et al., 2020; Liu et al., 2021). NMs enhance plant salt tolerance mainly by improving plant photosynthesis performance, promoting ROS detoxification, and maintaining ionic homeostasis and resoring osmotic balance (Newkirk et al., 2018; Liu et al., 2021). Compared with the non-nanoparticle control, the application of cerium oxide nanoparticles significantly improved cotton salt tolerance by b maintaining cytosolic Na^+^/K^+^ ratio (Liu et al., 2021). In addition, zinc oxide nanoparticles have been shown to enhanc salt tolerance in seedlings by improving photosynthetic pigments and antioxidative systems (Singh A et al., 2022). Remarkably, exogenous application of biocompatible poly (acrylic acid)-coated cerium oxide nanoparticles can improve the production of gasious signaling molecules (i.e., NO), therefore maintaining the redox and ionic homeostasis in rice under salt stress (Zhou et al., 2021). Although the underlying mechanisms need to be furtherly elucidated, nanobiotechnology could be a promising approach to increase crop yield in saline soils by enhancing plant salt tolerance.

## Concluding remarks and perspectives

Over the past decades, much progress has been made in understanding how plants respond and adapt to salt stress. Plants have evolved various regulatory mechanisms to cope with the damages caused by excessive saline ions in the soil. Osmotic adjustment, redox, and ionic homeostasis regulation, and metabolic adjustment are the significant factors associated with plant salt tolerance (**Figure 1**). To cope with salt stress, plants have to rapidly and effectively perceive changes in Na^+^ levels and osmotic pressure caused by salt stress. Different sensors mediate stress-signaling sensing, which relays stress signals to secondary messengers that activate signaling cascades and downstream regulatory networks *via* multiple hormone-mediated signaling pathways. The mechanisms of plant salt response involve a variety of signaling components, transcription factors, and functional genes that directly mediate ionic homeostasis, osmoregulation, and antioxidation (**Figures 2**, **3**). The phytohormone and light signals also mediate salt stress response in plants. Plant microbiota might also contribute to plant resistance. Exploring the molecular mechanisms of plant salt tolerance remains a great challenge. Many salt-responsive new genes still need to be annotated *via* advanced biotechnologies. The current knowledge of the salt-responsive molecular mechanisms in plants, from salt sensing and signaling to the development of adaptive tolerance mechanisms, still requires further studies. To date, the integration of multi-omics techniques and physiological phenotyping has proven to be a fast and effective method for probing the regulatory mechanism of plant salt tolerance (Song et al., 2020; Pazhamala et al., 2021). In particular, identification of upstream components regulating salt stress sensing is of paramount importance. Furthermore, the crosstalk between salt stress signaling networks and phytohormones still requires further investigation. Together, these findings provide valuable knowledge for breeding salt-tolerant crops through biotechnological approaches in the future.

## Author contributions

FX and HZ wrote this manuscript and prepared the illustrations. All the authors contributed to the discussion and agreed to the published version of the manuscript. All authors contributed to the article and approved the submitted version.
